# Synergistic Utilization of Recycled Asphalt Pavement and Fly Ash for High-Ductility Coal Mine Backfill: Performance Optimization and Mechanism Analysis

**DOI:** 10.3390/ma19020320

**Published:** 2026-01-13

**Authors:** Xiaoping Shao, Xing Du, Renlong Tang, Wei Wang, Zhengchun Wang, Yibo Zhang, Xin Gao, Shaofeng Hu

**Affiliations:** 1 College of Energy and Mining Engineering, Xi’an University of Science and Technology, Xi’an 710054, China; 2 Key Laboratory of Western Mines and Hazards Prevention, Ministry of Education of China, Xi’an 710054, China

**Keywords:** recycled asphalt pavement, backfill, fly ash, ductility, solid waste treatment

## Abstract

**Highlights:**

**What are the main findings?**

- RFCPB slurry follows H-B model, with rheology regulated by F/B ratio.
- 28-day UCS of RFCPB varies with F/B ratio (first rise, then fall).
- RAP enhances backfill ductility, realizing brittle-to-ductile transition.
- RAP’s Dolomite/Albite synergizes with FA to generate gels, optimizing pore structure and UCS.

**What are the implications of the main findings?**

- RFCPB meets pipeline transportation and mine backfill technical requirements.
- Brittle-to-ductile transition mitigates goaf collapse risk, protecting overlying highways.
- Clarifies RAP-FA chemical synergy in cement-based composites, filling knowledge gaps.

**Abstract:**

To enhance the ductility of coal mine filling materials using recycled asphalt pavement (RAP) and address the limitations in RAP recycling and utilization, this study processed RAP into crushed materials (CMs) and ball-milled materials (BMs). Supplementary with fly ash (FA) and cement, RAP-fly ash cement paste backfill (RFCPB) was prepared. For 1000 g of RFCPB slurry, the composition was 365 g CM, 73 g cement, 270 g water, and a total of 292 g of FA and BM, with an F/B ratio ranging from 1:7 to 7:1. A systematic test program was carried out, including rheological property tests, unconfined compressive strength (UCS) tests combined with deformation monitoring, microstructure analysis, and leaching toxicity tests. Based on these tests, the influence of F/B ratio on the action mechanism, workability, mechanical properties, ductility and environmental compatibility of RFCPB was comprehensively explored. The results show that the rheological behavior of RFCPB slurry conforms to the Herschel–Bulkley (H-B) model; with the decrease in F/B ratio, the yield stress and apparent viscosity of the slurry increase significantly, while the slump and slump flow decrease correspondingly, which is closely related to the particle gradation optimization by BM. For mechanical properties and ductility, the 28-day UCS of RFCPB first increases and then decreases with the decrease in F/B ratio, all meeting the mine backfilling strength requirements; notably, the increase in BM proportion regulates the failure mode from brittle to ductile, which is the key to improving ductility. Microstructural analysis indicates that Dolomite and Albite in BM participate in hydration reactions to generate N-A-S-H and C-A-S-H gels, which fill internal pores, optimize pore structure, and thus synergistically improve UCS and ductility. Additionally, the leaching concentration of toxic ions in RFCPB complies with the environmental protection standards for solid waste. This study provides a theoretical basis for enhancing backfill ductility and advancing the coordinated disposal of RAP and fly ash solid wastes.

## 1. Introduction

Coal is an important primary energy source in China, with an annual output that reached approximately 4.759 billion tons of standard coal in 2024 [1]. It is projected to account for 45% of China’s primary energy consumption in 2030 [2]. Goafs formed by mining activities in coal mines are prone to inducing surface subsidence and ecological degradation, which not only threaten the operational safety of existing highways but also restrict the planning and implementation of new highway projects [3]. Goaf backfilling technology is a key technical pathway to realize the coordinated development of mine ecological restoration and highway engineering. Currently, cement-based composite backfill (CBC: a mixture composed of hydraulic cement, mining waste, and mixed water) is widely adopted in mine backfilling operations [4,5,6]. CBC can assist the mining industry in efficiently recovering ore and water resources, shortening the mining cycle, reducing environmental pollution, and lowering production costs; thus, its importance in mining engineering has become increasingly prominent [7,8,9]. However, after cement hydration, CBC exhibits characteristics of high brittleness and low ductility, posing potential risks to the safety of operating personnel and equipment [10]. Appropriately enhancing ductility enables the backfill to form a flexible contact interface with surrounding rock, which helps alleviate the dynamic pressure of overlying strata, adjust the stress state of surrounding rock, and avoid cracking caused by insufficient tensile strength [11,12,13,14].

Currently, the mainstream technical approach to enhancing the ductility of backfill materials is fiber modification within the CBC system [15]. Cao et al. [16] investigated the effects of polypropylene fiber, polyacrylonitrile fiber, and glass fiber on the strength, toughness, and microstructure of early-age cement tailings backfill (CTB). The findings indicated that the type and content of fibers significantly regulate the ductility of CTB, with strength increasing linearly with fiber content and crack resistance being remarkably improved. To address the problem of high brittleness and poor toughness of cemented gangue backfill, Che et al. [17] incorporated waste tire steel fiber (WTSF) with different dosages into the backfill system, which achieved the transformation of the backfill from brittle to ductile behavior. The toughening mechanism is attributed to the interlocking and bridging effects of the fibers. However, such fiber modification methods generally suffer from drawbacks including high cost, strict requirements for backfilling processes, and high carbon emission intensity [18,19,20]. Thus, the development of low-cost, sustainable alternative schemes for high-ductility backfill materials has become a core technical challenge urgently needing to be addressed in the field of mining engineering.

Fly ash (FA) is a by-product generated from the combustion of coal in thermal power plants or industrial boilers [21]. In 2024, China’s FA production is estimated to reach approximately 930 million tons [22]. The pozzolanic activity of FA contributes to the long-term strength development of backfill materials, while its spherical particle morphology (microsphere effect) enhances the fluidity of the backfill slurry. Thus, partially replacing cement with FA can significantly optimize the overall performance of the backfill [23,24,25,26]. Conversely, the large-scale construction and maintenance of highways, a critical component of the national economy, have generated substantial amounts of recycled asphalt pavement (RAP). China’s annual RAP production reaches approximately 200 million tons [27,28]. Currently, the disposal of reclaimed asphalt pavement (RAP) is predominantly dependent on reclaimed asphalt recycling technology [29,30]. Though it has achieved certain practical application results, significant limitations remain—low recycling rate (<30%) [31,32,33], complex milling process [34], and the requirement for secondary modification of recycled product performance [35,36]—which hinder the efficient resource utilization of substantial quantities of RAP. Notably, the aged asphalt contained in RAP naturally exhibits ductile properties. Chen et al. [37] demonstrated that the ductility of conventional base asphalt exceeds 100 mm, and even after aging, its ductility can still reach 14.6 mm at 15 °C. Thus, the synergistic application of fly ash (FA) and RAP in mine backfill material systems can not only address the technical bottleneck of CBC’s high brittleness but also realize the large-scale absorption of two types of bulk solid wastes, featuring technical feasibility, resource recycling value, and environmental benefits.

Currently, researchers have extensively investigated the application of FA and RAP in road base materials. Rachita et al. [38] demonstrated that a blend of FA and RAP, as a replacement for traditional pavement base materials, exhibits satisfactory physical properties, compressive strength, elastic modulus, and durability, all meeting the requisite standards. Mohammadinia et al. [39] reported that FA, utilized as a low-carbon stabilizer, significantly enhances the road performance of recycled aggregate. Their findings indicated that a 15% FA content yielded the highest UCS and elastic modulus under curing conditions at both room temperature and 40 °C. Horpibulsuk et al. [40] investigated RAP and FA as sustainable pavement materials. They observed that the high calcium content in RAP reacted with the abundant silica and alumina in FA, leading to the formation of calcium silicate hydrate (C-S-H), thereby enhancing the strength of the RAP-FA blends. Hoy et al. [41] reported that in RAP-FA geopolymers, an N-A-S-H gel formed under alkaline activation and coexisted with the C-S-H and C-A-S-H gels produced from the reaction of RAP and FA. This synergistic gel formation significantly increased the UCS, confirming the feasibility of utilizing this material as a low-carbon road base [38]. Although existing studies have confirmed the mechanical property advantages and reaction mechanisms of the synergistic effect between fly ash (FA) and reclaimed asphalt pavement (RAP), their application scenarios are still limited to pavement base courses. Mine backfill materials are required to withstand the surrounding rock pressure of goafs and meet special service requirements such as deformation resistance and long-term stability, leading to essential differences in performance demands between mine backfill and pavement base courses [42,43,44]. Therefore, systematic research on the applicability, synergistic mechanism, and performance regulation laws of the FA-RAP synergistic system as a mine backfill material is currently lacking, and the relevant research gap urgently needs to be addressed.

To synergistically address the dual challenges of insufficient stability of traffic highways overlying goafs and low utilization efficiency of RAP, while promoting sustainable and green development, this study innovatively proposes and develops a RAP-FA cement paste backfill (RFCPB) material. The RFCPB is composed of recycled RAP, fly ash (FA), cement, and water, with recycled RAP as the core base material. The core innovations of this study are summarized as follows: (1) The synergistic utilization of mine solid waste (FA) and highway solid waste (RAP) is achieved, and a novel strategy for enhancing backfill stability by leveraging the inherent ductility of RAP is proposed. (2) For the first time, the majority of RAP is utilized as aggregate after crushing, while a fraction is ball-milled into a fine powder. The feasibility and underlying mechanisms influencing the performance of RAP in both aggregate and powder forms are comprehensively investigated. (3) The effects of varying FA/BM mass ratios on the rheology, strength development, deformation characteristics, and long-term stability of RFCPB are comprehensively investigated.

## 2. Materials and Methods

### 2.1. Material

**Figure 1:**
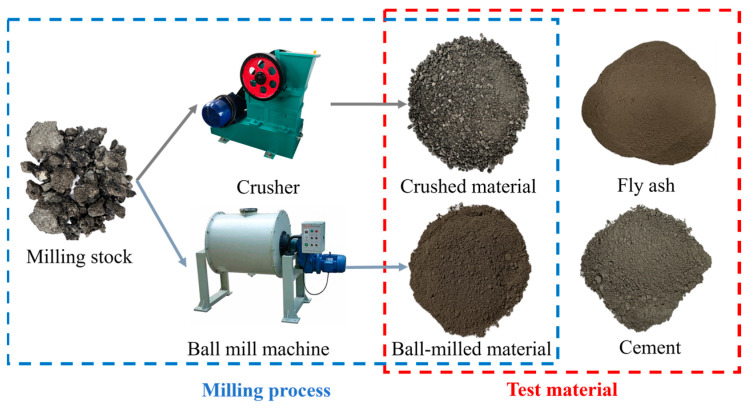
Test material.

Raw materials included recycled asphalt pavement (RAP), Class I fly ash (met the requirements of GB/T 1596-2017 [45]) from the Yulin Jinjie Coal-fired Power Plant, and Ordinary Portland cement (P.O 42.5) as the binder; tap water from Xi’an municipal water supply was used for mixing. RAP was sourced from highway maintenance waste (complying with GB/T 25033-2010 [46]) and crushed into crushed material (CM) via a jaw crusher, with particle size controlled at 2.36–9.5 mm and impurity content ≤ 1.5%; the remaining RAP was ball-milled into ball-milled material (BM) using a planetary ball mill (400 r/min, 2 h), with ≥90% passing through a 0.3 mm sieve. The physical appearances of raw materials are presented in Figure 1.

The chemical compositions of the raw materials were accurately determined via X-ray Fluorescence (XRF) spectroscopy, with the test results summarized in Table 1. As illustrated, the base material (BM) is predominantly composed of SiO_2_ and CaO, while fly ash (FA) is mainly constituted by SiO_2_, and ordinary Portland cement (OPC) is dominated by CaO.

**Figure 2:**
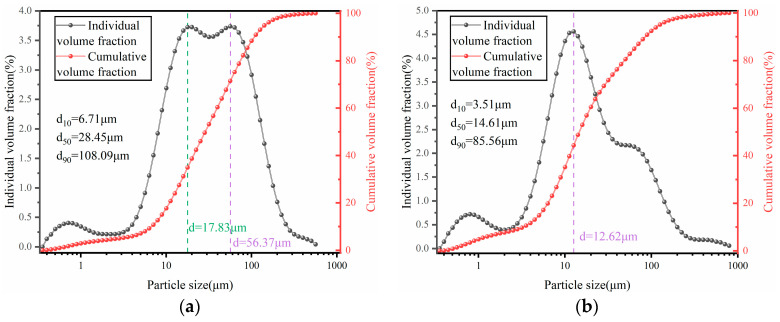
Particle size distribution of BM and FA: (**a**) BM; (**b**) FA.

The particle size distribution (PSD) of the raw materials is presented in Figure 2. From the cumulative distribution curve, FA exhibits the smallest D_50_ value (14.61 μm), with the cumulative volume fraction of fine particles (<10 μm) reaching 35.2%—significantly higher than that of BM (17.7%), indicating a higher content of fine particles in FA. In contrast, BM possesses the largest D_90_ value (108.09 μm) and a higher proportion of coarse particles, resulting in an overall rightward shift in its cumulative distribution curve. Regarding the volume frequency distribution curve, FA shows the smallest peak particle size (12.62 μm) and a sharper peak shape, suggesting a more concentrated particle size distribution. Conversely, BM exhibits the largest peak particle size and a gently transitioning bimodal distribution, with the two peaks centered at 17.83 μm and 56.37 μm, respectively, indicating a more uniform particle size distribution for BM.

**Figure 3:**
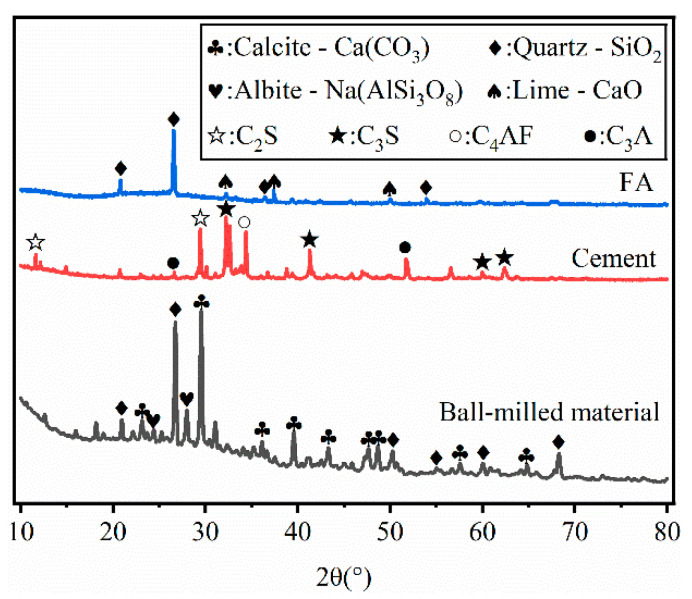
XRD of BM, Cement, and FA.

The X-ray diffraction (XRD) patterns of the raw materials are displayed in Figure 3. It can be observed that BM is primarily composed of CaCO_3_, SiO_2_, and Na(AlSi_3_O_8_), while FA mainly consists of SiO_2_ and CaO. OPC, on the other hand, is characterized by the presence of major mineral phases including C_2_S, C_3_S, C_4_AF and C_3_A.

**Figure 4:**
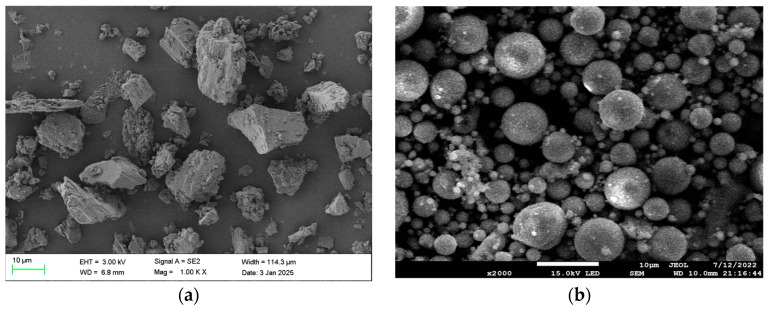
SEM image of BM and FA: (**a**) BM; (**b**) FA.

The micro-morphologies of the raw materials are shown in Figure 4. BM particles exhibit an irregular polygonal shape with rough and uneven surfaces, accompanied by large interparticle pores. In contrast, FA particles present a smooth spherical morphology and a densely packed arrangement.

### 2.2. Backfill Preparation

Preliminary experiments confirmed the backfill solid mass concentration of 73%, with cement accounting for 10% of the total solid mass; F/B denotes the FA-to-BM ratio, and the dosages of F/B, FM, CM and cement are listed in Table 2.

**Figure 5:**
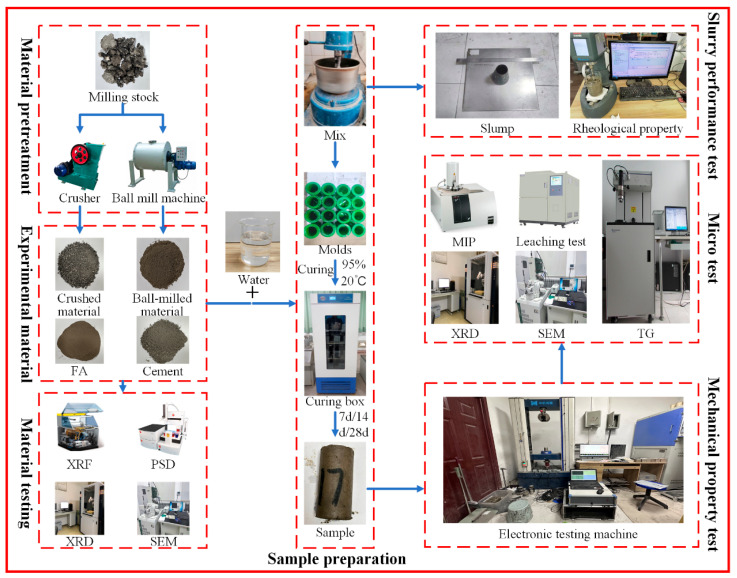
Experimental process.

After verifying raw material quality, RFCPB slurry was mixed using a JJ-5 cylindrical concrete mixer: dry materials (CM, BM, FA, cement) were pre-stirred for 2 min, followed by slow water addition and 3 min stirring until lump-free, then left to stand for 30 s for defoaming. The defoamed slurry was immediately poured into 70 × 70 × 70 mm standard cubic molds, smoothed, and initially cured in a constant temperature-humidity chamber (20 ± 2 °C, RH ≥ 95%). Demolding was conducted after 24 h, and specimens were continuously cured in the same chamber to preset ages (7, 14, 28 d) [47]. The experimental flowchart is shown in Figure 5.

### 2.3. Methods and Standards

#### 2.3.1. Flow Performance Test

After the mixing of RFCPB filling slurry, the rheological properties of the slurry were quantitatively characterized by the Viscotester iQ rotary rheometer (Thermo Fisher Scientific, Waltham, MA, USA), and the interaction force and shear response behavior between particles in the slurry were revealed.

In this experiment, a slump tester was used to conduct the slump test. The height of the central highest point and the diffusion diameter of the slurry were measured, and the slump and slump flow were calculated accordingly. According to GB/T 50080-2002, the flowability and pumping performance of the filling slurry are quantitatively evaluated [48].

#### 2.3.2. Mechanical Property Measurement

Under the condition that the RFCPB samples reached the curing ages (7 d, 14 d, 28 d), the UCS tests were carried out using the Sino Test DNS100 microcomputer-controlled (Sino Test Co., Ltd., Changchun, China) electronic universal testing machine according to GB/T 50081-2019 at a quasi-static loading rate of 1.0 mm/min. All experiments were conducted in duplicate, and the average UCS value was calculated from the test results [47].

#### 2.3.3. Microstructure Test

The TGA5500 thermogravimetric analyzer (NETZSCH, Selb, Germany) was used to determine the mass change behavior of RFCPB samples as a function of curing time (7 d, 14 d and 28 d) under programmed temperature control, and the thermal stability, decomposition temperature and residue content of the samples were analyzed. First, the sample was ground to powder using a mortar and placed in a crucible. The crucible was suspended from the balance arm, and then the furnace body was closed and sealed. Nitrogen was introduced to purge the residual oxygen in the furnace. Finally, a temperature program was employed to heat the system from 25 °C to 1000 °C at a rate of 10 °C/min, and TG and DTG were recorded in real time.

The samples were characterized using a Bruker D8 ADVANCE X-ray diffractometer (Bruker AXS SE, Karlsruhe, Germany). After the UCS test of the backfill specimens, powder samples were first drilled from the middle of the backfill and placed in anhydrous ethanol to terminate the hydration reaction. Then, the characteristic peak intensity was tested and recorded at a scanning rate of 5°/min and a spectral range of 10–80°. Finally, JADE 6.0 analysis software was used to perform automatic peak searching [49].

The microstructure of the backfill was examined using a JEOL JSM-6460LV low-vacuum scanning electron microscope (JSM-7610F, Akishima-shi, Japan). After the UCS test, a thin section with a thickness of approximately 2 mm was cut from the central area of the backfill, and a 10 nm thick gold film was sprayed on the surface of the sample to enhance electrical conductivity [50].

#### 2.3.4. Leaching Toxicity Test

The samples were crushed to a particle size ≤9.5 mm, and the extractant was added at a liquid-solid ratio of 20:1 (L/kg). The mixture was placed in a horizontal shaker (30 ± 2 °C) and shaken at a constant temperature (23 ± 2 °C) for 18 ± 2 h. The Solid Waste Leaching Toxicity Leaching Method (HJ 557-2010) was employed for the evaluation. The toxicity leaching test aims to evaluate the leaching risk of hazardous substances in solid waste in a simulated natural environment and determine whether such leaching poses environmental harm [51].

## 3. Results and Discussion

### 3.1. Flow Characteristics of RFCPB

#### 3.1.1. Rheological Characteristics of RFCPB

In this experiment, the Herschel–Bulkley (H-B) model was employed to characterize the rheological properties of the RFCPB filling slurry. The H-B model can provide the relatively most stable estimation of dynamic yield stress [52]. The H-B model is mathematically expressed as [53]:

(1) $$\tau =\tau_{0}+K\gamma^{n}$$

where τ is shear stress, Pa; $\tau_{0}$ is yield stress, Pa; K is the plastic viscosity coefficient, Pa·s; γ is the shear rate, s^−1^; n is the rheological property index.

The fitting results of the H-B model to the rheological test data of RFCPB slurries showed that the coefficient of determination R^2^ of all experimental groups was greater than 0.99, which confirmed that the model was applicable for characterizing the rheological behavior of RFCPB slurries in this study. Table 3 lists the key rheological parameters of RFCPB slurry obtained by H-B model fitting. It is worth noting that the rheological index n of the slurry at all ratios is greater than 1. According to [54], this observation indicates that the slurries exhibit shear thickening behavior within the tested shear rate range, which has potential benefits for sustaining the stability of the slurries during pipeline transportation.

**Figure 6:**
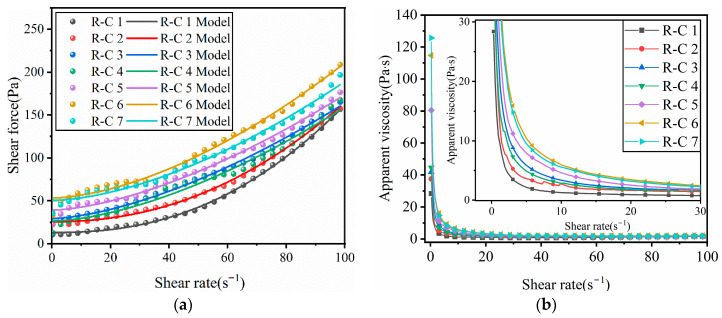
Rheological properties of RFCPB slurry: (**a**) The relationship between shear rate and shear force; (**b**) The relationship between shear rate and viscosity.

Figure 6a shows the relationship between the shear stress of RFCPB slurries and the applied shear rate under different F/B ratios; overlaid on this figure are the H-B model fitting curves derived from the rheological parameters presented in Table 3. It can be observed that the shear stress of all RFCPB slurries increased monotonically with increasing shear rate. This phenomenon can be attributed to the fact that as the applied shear rate increases, the internal friction between the particles and flocculated structures increases, leading to an increase in the macroscopically measured shear stress. As shown in Table 3 and Figure 6a, the dynamic yield stress of RFCPB slurries increased significantly with increasing F/B ratio. This phenomenon can be mainly attributed to two mechanisms: (1) FA particles are usually spherical in shape, while BM particles are irregular. FA particles can exert a ‘ball-bearing effect’ in the slurries, and this effect intensifies as FA content increases, thus reducing the flow resistance of the slurries. This observation is consistent with the conclusions of Zhang et al. [55] and Yang et al. [56]. (2) The proportion of FA particles in the 1–10 μm range is higher than that of cement particles. The incorporation of FA can optimize the particle size distribution of the composite cementitious system and increase the particle bulk density. This leads to the relative release of free water originally filling the gaps between cement particles, which in turn improves the fluidity of the slurries and aids in mitigating the shear thickening effect [55]. At the same time, FA fills the gaps of coarse particles to exert a lubricating effect, which can reduce the frictional resistance between particles [57]. In contrast, BM particles are mainly distributed in the 10–20 μm range, with a small fraction in the 1–10 μm range. Their contribution to optimizing particle gradation and increasing bulk density is relatively limited, so the enhancement of slurry fluidity is relatively modest.

Figure 6b shows the relationship between shear rate and apparent viscosity under different F/B ratios. It can be clearly observed from the figure that the viscosity increases with decreasing F/B ratio, and the apparent viscosity of each RFCPB slurry decreases with increasing shear rate, which indicates that the slurries have shear thinning characteristics. In addition, it is further observed in Table 3 that the apparent viscosity of the RFCPB slurries is consistent with the rheological index n values, both of which exhibit shear thinning characteristics [58].

#### 3.1.2. Slump and Slump Flow of RFCPB

**Figure 7:**
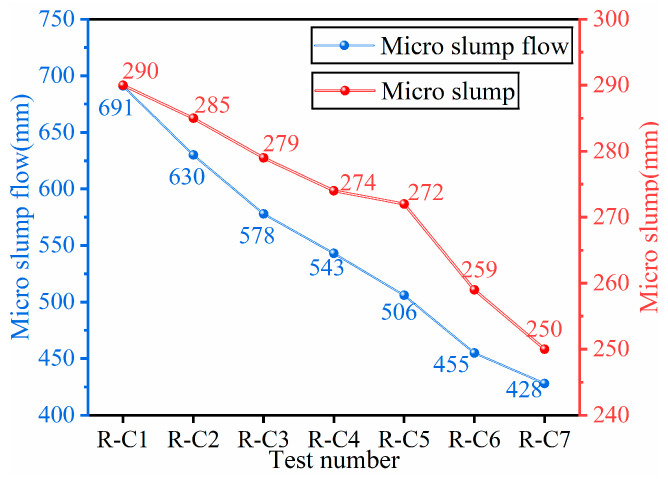
Slump and slump flow characteristics of RFCPB slurry.

Figure 7 shows the test results of slump and slump flow of RFCPB filling slurries under different F/B ratios. The results showed that with decreasing F/B ratio, the slump of the filling slurries exhibited a continuous decreasing trend. The measured slump values of Groups R-C1 to R-C7 were 290 mm, 285 mm, 279 mm, 274 mm, 272 mm, 259 mm and 250 mm, respectively. It is worth noting that a significant acceleration in the slump reduction was observed in Group R-C6, which was 141% higher than the average decrease in the other groups. According to [59], a slump value exceeding 71 mm indicates that a filling slurry has good pumpability and satisfies the requirements for pipeline transportation. Therefore, all the RFCPB ratios tested in this study satisfy the slump requirements for coal mine backfilling. The expansion diameter of the filling slurries was consistent with the slump, and it also exhibited a continuous decrease with decreasing F/B ratio. The expansion diameter values were 691 mm, 630 mm, 578 mm, 543 mm, 510 mm, 455 mm and 428 mm for Groups R-C1 to R-C7. The expansion diameter also exhibited a significant deterioration in Group R-C6, and its decrease was 32% higher than the average decrease in the preceding groups. According to [60], an expansion diameter exceeding 220 mm indicates that a slurry has good fluidity. The continuous decrease in the slump and slump flow of RFCPB slurries for Groups R-C1 to R-C7 can be attributed to the rougher surface, more irregular shape, and larger specific surface area of BM [61]. These characteristics lead to an increase in the water adsorption capacity of BM, thus reducing the free water content in the system, thereby reducing the fluidity of the slurries. Comprehensive analysis results demonstrate that the slump and slump flow of all filling slurries are consistent with the performance criteria for excellent fluidity. Nevertheless, significant deterioration behaviors were observed in the R-C6 and R-C7 groups. Consequently, it is advisable to prioritize the selection of formulations within the R-C1 to R-C5 groups for subsequent applications.

### 3.2. Mechanical Characteristics of RFCPB

#### 3.2.1. Uniaxial Compressive Strength of RFCPB

The RFCPB samples were prepared according to the mix ratios specified in Section 2.2. After curing at the specified curing ages, UCS tests were performed using an MTS electronic universal testing machine. Table 4 summarizes the average UCS values, standard deviation (SD), coefficient of variation (CV), 95% confidence interval (95% CI) and significance test results of each mix ratio sample at different curing ages [62]. The test results showed that the standard deviation (SD) of UCS values of all samples was less than 0.2, and the coefficient of variation (CV) was generally less than 10% (only R-C5 at 7 d was 10.48%), indicating that the test data had good reliability and consistency. Additionally, one-way ANOVA revealed extremely significant differences in UCS among different mix ratio samples at each curing age (*p* < 0.001), while repeated measures ANOVA confirmed a highly significant increase in UCS with curing time (*p* < 0.001). The narrow 95% CIs of all samples further verified the high accuracy of the mean value estimation, enabling the data to be used for subsequent analysis.

**Figure 8:**
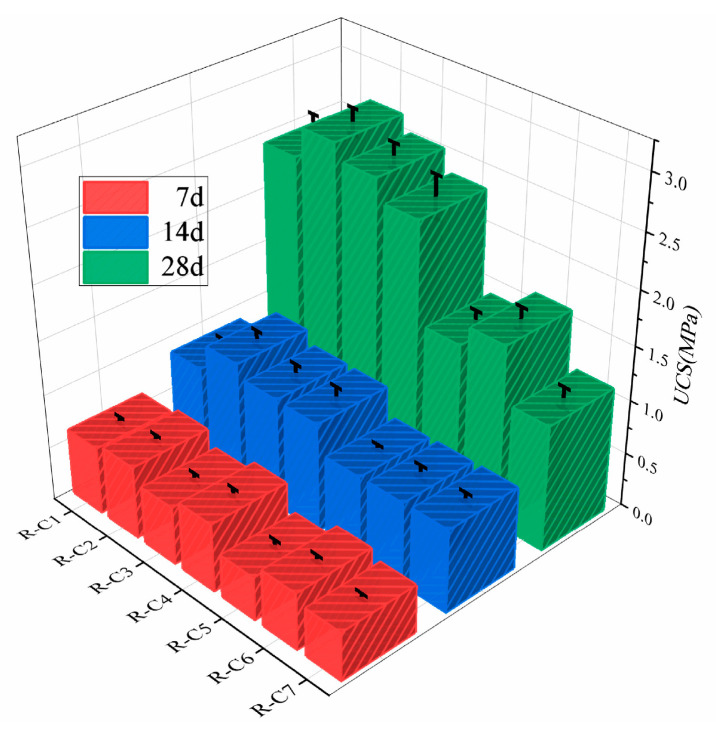
RFCPB UCS values for different curing ages.

Taking Group R-C4 as an example, Figure 8 shows that the UCS values increased significantly with increasing curing age, from 0.638 MPa at 7 d to 1.020 MPa at 14 d, and further reached 2.389 MPa at 28 d. This increase in strength is mainly attributed to the continuous hydration reactions that generate a large number of hydration products, which effectively fill the pores within the backfill material, thereby improving the backfill’s compactness and overall compressive strength. This trend is consistent with the research conclusions of Guan et al. [63].

At 28 days of curing, the UCS values of RFCPBs increased first and then decreased as the F/B ratio varied. Specifically, the samples in Group R-C2 (F/B ratio = 6/2) exhibited the highest UCS values. The strength improvement can be attributed to the synergistic effect of FA and BM: the active components in FA participate in the pozzolanic reaction, consume the Ca(OH)_2_ produced by cement hydration and generate additional gel phases such as C-S-H (calcium silicate hydrate); at the same time, the incorporation of an appropriate amount of BM can also form hydration products, further increasing the total amount of hydration products and the density of the matrix. However, when the F/B ratio decreased to 3/5 or lower, the UCS values decreased significantly. Compared with Group R-C4, the strength of Group R-C5 decreased by 38.4%, which was greater than the average decrease of 11.1%, indicating that the content of BM exceeded its critical threshold content. This is because the hydraulic reactivity of BM is usually lower than that of FA and cement, and excessive BM dilutes the concentration of the highly active cementitious components in the system. Suksun et al. [64] noted that the essence of cementation strength stems from the dense, continuous three-dimensional cementitious network formed between cementitious materials and aggregates. In this study, the backfills from Groups R-C1 to R-C4 exhibited relatively higher 28-day UCS values, indicating that a more well-developed and effective cementitious structure system was formed inside. In coal mine backfill mining, the 28-day UCS value of the backfill is typically required to range between 0.7 and 2.0 MPa to ensure the safety and stability of the mine [65].

#### 3.2.2. Stress–Strain Curve Characteristics of RFCPB

**Figure 9:**
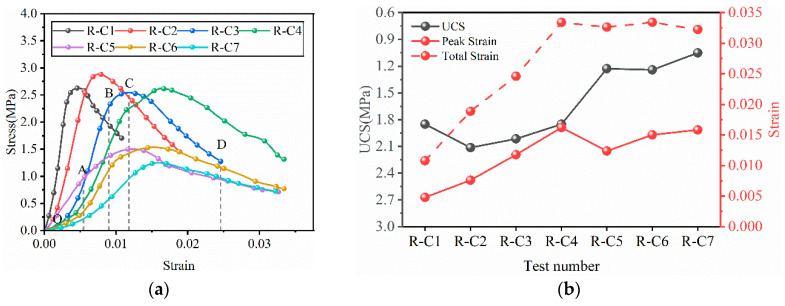
Stress–strain curve, UCS and strain of RFCPB: (**a**) Stress–strain curve, which can be divided into four stages according to their deformation characteristics: pore and fissure compaction stage (OA), elastic deformation stage (AB), plastic deformation and yield stage (BC), and post-peak softening and failure stage (CD); (**b**) UCS and strain.

At an applied loading rate of 1 mm/min, the typical uniaxial compressive stress–strain curves of RFCPBs with different F/B ratios are presented in Figure 9a. According to their deformation characteristics, the curves can be divided into four stages [66]: pore and fissure compaction stage (OA), elastic deformation stage (AB), plastic deformation and yield stage (BC), and post-peak softening and failure stage (CD).

The pore and fissure compaction stage (OA): At this initial stage, the stress–strain curves of all RFCPB specimens exhibit an upwardly concave characteristic. It is worth noting that as the F/B ratio decreases, this upwardly concave feature becomes increasingly pronounced. This phenomenon is mainly attributed to the fact that BM particles are mainly distributed in the 17–56 μm range, endowing them with a large specific surface area and high water adsorption capacity. In the presence of water, the adsorbed water layer formed around the BM particles may lead to a relative increase in the volume of internal microcracks within the backfill material. During the compaction stage, the closure of these cracks requires higher strain, manifesting as a more pronounced upwardly concave curve.

Elastic deformation stage (AB): At this stage, the stress–strain relationship is approximately linear. At this stage, most of the input energy is converted into elastic strain energy and stored in the specimens [67,68]. As the F/B ratio decreases, the slope of the curves in the AB section gradually decreases, and the linear deformation stage becomes shortened.

Plastic deformation and yield stage (BC): The curves at this stage exhibit an upwardly convex shape. With the accumulation of plastic deformation, the elastic strain energy stored in the specimens decreases, and energy dissipation increases markedly. This corresponds to the initiation and stable propagation of microcracks in the RFCPBs until the stress reaches its peak value. With decreasing F/B ratio, the strain magnitude required for the specimens to reach the peak stress gradually increases.

Post-peak softening and failure stage (CD): At this stage, the stress decreases with increasing strain, indicating that the material undergoes unstable failure. The internal microcracks propagate rapidly and interconnect, eventually leading to macroscopic damage [69]. With decreasing F/B ratio, the total strain required for the specimens to undergo macroscopic failure increases significantly.

The uniaxial compressive strength and strain characteristics of different RFCPBs are presented in Figure 9b. It illustrates that as the F/B ratio decreases, the total strain and peak strain increase steadily from R-C1 to R-C4, while the UCS values remain at a high level. From R-C4 to R-C7, UCS values decreased markedly. The total strain tends to stabilize after peaking in Group R-C4, while the peak strain decreases suddenly in Group R-C5. These strain variations indicate that the ductility of RFCPBs is optimal for Group R-C4 [70]. For the first four groups, as the F/B ratio decreases, the structure of RFCPBs becomes more stable; that is, when RFCPBs are subjected to high pressure, the deformation rate is accelerated, and the internally accumulated energy is quickly released. Therefore, they require more time and greater displacement to fail when subjected to pressure [71].

#### 3.2.3. Macroscopic Failure Mode of RFCPB

**Figure 10:**
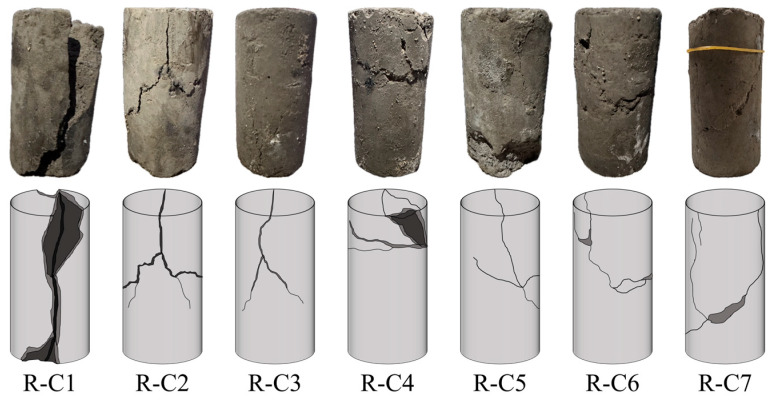
Uniaxial compression failure mode.

The uniaxial compression failure modes of RFCPB samples with different F/B ratios after 28 days of curing are presented in Figure 10. Observations indicate that as the F/B ratio decreases, the failure modes of RFCPBs gradually transition from brittle splitting tensile failure to ductile shear failure. Typical brittle failure modes include axial splitting, crushing failure, and conical failure. Ductile failure modes are characterized by significant bulging deformation, formation of clear shear bands, and continuous deformation without a distinct macroscopic fracture surface [72].

Group R-C1 (high F/B ratio) mainly exhibits a single main shear failure surface extending through the specimen, and the failure location is concentrated in the middle of the specimen [73]. This failure mode can be attributed to the fact that during the initial loading stage, stress concentration on the top and bottom surfaces of the specimen exceeds the local strength due to the contact effect, which causes damage that propagates toward the center, resulting in large compressive deformation of the entire specimen. At the same time, under axial compression, the tensile stress induced in the radial direction of the specimen exceeds the tensile strength of the intergranular bonds, and finally results in a splitting crack throughout the entire specimen axis [74,75]. This failure mode is a typical splitting failure, which is consistent with the previous findings of Liu et al. [76]. For Groups R-C2 and R-C3 (medium F/B ratio), the failure modes evolved into conjugate shear failure, and the failure locations were mainly concentrated in the middle and upper parts of the specimens [77]. For Groups R-C4 to R-C7 (low F/B ratio), as BM content further increases, the failure modes gradually changed to axial splitting failure, and the failure locations were mostly concentrated in the middle of the specimens. A notable characteristic is that the specimens exhibit strong lateral expansion deformation, forming multiple non-penetrating cracks. Although the specimens are cracked, they still retain a degree of integrity, and connections exist between the blocks. This characteristic aligns with the definition of ductile failure modes [69,78]. Zhang et al. [79] noted that the incorporation of FA usually does not change the brittle failure mode of backfills. Combined with the observations from this study, it can be clearly deduced that increasing BM content is the key factor leading to the transition in failure modes of RFCPBs from brittleness to ductility.

### 3.3. Microscopic Characteristics of RFCPB

#### 3.3.1. TG Characteristics of RFCPB

**Figure 11:**
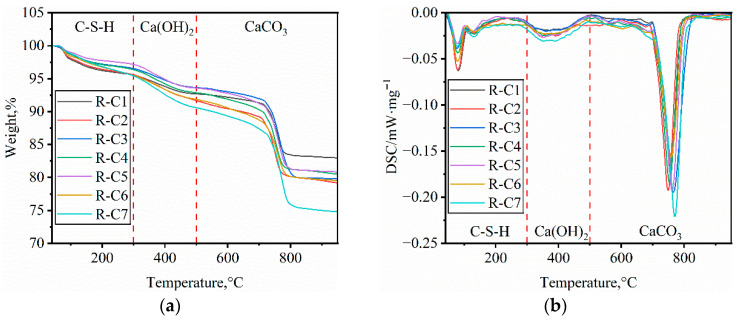
TG-DTG results of curing 28d RFCPB samples: (**a**) TG; (**b**) DTG.

To investigate the main phase composition of RFCPBs after 28 days of curing, the samples were characterized by thermogravimetric analysis. Figure 11 shows the TG and DTG curves for RFCPB samples. TG was used to determine the mass loss corresponding to the thermal decomposition of different phases in the backfills, while DTG was used to identify the corresponding thermal decomposition temperature ranges of different phases in the paste matrix [80]. Figure 11a shows that all the samples exhibited continuous mass loss behavior during heating. Figure 11b further reveals three significant weight loss stages, corresponding to the thermal decomposition reactions of specific phases in different temperature ranges, which are consistent with the previous findings of Wang et al. [81].

The first stage (room temperature-300 °C): The weight loss at this stage mainly involves three consecutive processes, specifically, 40–100 °C, where weight loss is primarily attributed to the evaporation and desorption of physically adsorbed water and partial free water; 110–170 °C, corresponding to the decomposition of ettringite (AFt); and 180–300 °C, where weight loss is mainly due to the gradual removal of C-S-H gel structural water. The weight loss behavior of each phase aligns with the observations of Tang et al. [82].

The second stage (300–500 °C): The steep DTG endothermic peak observed in this interval is mainly attributed to the dehydration of Ca(OH)_2_. Its peak temperature is highly consistent with the characteristic decomposition temperature of Ca(OH)_2_, confirming the presence of the cement hydration product Ca(OH)_2_, a conclusion corroborated by Wang et al. [83].

The third stage (500–900 °C): This stage is characterized by a broad endothermic peak. The significant weight loss peak in the DTG curve at 760 °C corresponds to the thermal decomposition of calcite (CaCO_3_), which is consistent with the findings of Yao et al. [84].

Figure 11a illustrates the mass loss extent of RFCPBs of different mix ratios, with total mass loss rates of 17.03%, 20.83%, 20.22%, 19.49%, 19.17%, 20.46% and 25.22%, respectively. Group R-C7 exhibited the highest mass loss, as FA content was insufficient to fully react with Ca(OH)_2_, and the unreacted Ca(OH)2 was partially converted into CaCO3 [85].

#### 3.3.2. XRD Characteristics of RFCPB

**Figure 12:**
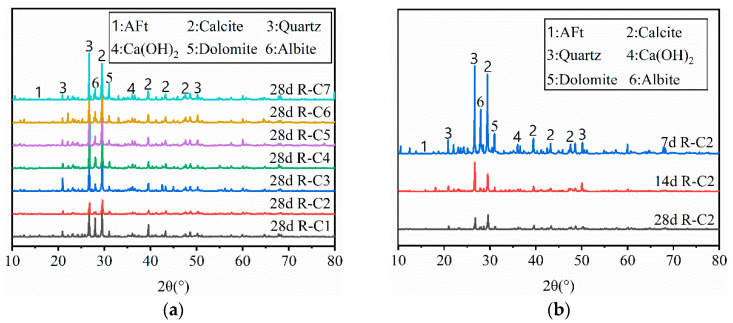
XRD patterns of RFCPB: (**a**) XRD patterns of RFCPB cured for 28d; (**b**) XRD patterns of R-C2.

The X-ray diffraction (XRD) patterns of RFCPBs are presented in Figure 12, revealing the evolution of typical phases under different F/B ratios and curing ages. The main crystalline minerals detected include quartz (SiO_2_), calcite (CaCO_3_), ettringite (AFt), Ca(OH)_2_, and the RAP characteristic minerals dolomite (CaMg(CO_3_)_2_) and albite (Na(AlSi_3_O_8_)). In RFCPB slurries, cement first reacts with water to consume gypsum, C_2_S, and C_3_S, generating hydration products such as AFt, C-S-H, and Ca(OH)_2_ [86]:

Subsequently, the active SiO_2_ and Al_2_O_3_ in FA react with Ca(OH)_2_ to form C-S-H gel and hydrated calcium aluminate [87]:

(2) $${\mathrm{Al}}_{2}O_{3}(s)\;+\;\mathrm{Si}O_{2}(s)\;+\;4H_{2}O\;+\;3{\mathrm{OH}}^{-}\left(\mathrm{aq}\right)\to {2\mathrm{Al}(\mathrm{OH})}_{4}^{-}{+\;\mathrm{Si}(\mathrm{OH})}_{3}^{-}$$

Among them, C-S-H, N-A-S-H and C-A-S-H gels has an amorphous structure, which is difficult to detect via XRD [88]. Thus, the following analysis is based on the variation in crystalline precursor minerals to infer the possible formation of such gel products.

Figure 12a shows that as the F/B ratio decreases, the diffraction peak intensity of quartz, calcite, AFt, Ca(OH)_2_, dolomite, and albite exhibits a trend of first decreasing and then increasing. This indicates that replacing FA with an appropriate amount of BM can gradually promote the hydrolysis of dolomite and albite in an alkaline environment, with their reduced diffraction peak intensity suggesting the formation of gel products (possibly N-A-S-H and C-A-S-H gels) through related reactions:

(3) $${\mathrm{NaAlSi}}_{3}O_{8}{+\;4H}_{2}O+{\mathrm{OH}}^{-}\to {\mathrm{Na}}^{+}{+\;\mathrm{Al}(\mathrm{OH})}_{4}^{-}{+\;3\mathrm{SiO}}_{3}^{2-}$$

(4) $${\mathrm{CaMg}(\mathrm{CO}}_{3}{)}_{2}{+4H}_{2}O\to {\mathrm{Ca}(\mathrm{OH})}_{2}{+\mathrm{Mg}}^{2+}{+2\mathrm{HCO}}_{3}^{-}$$

This is consistent with the previous findings of Rachita et al. [38]. In their study, FA and RAP were employed to replace the cementitious materials of traditional concrete, and it was found that Na(AlSi_3_O_8_) and CaMg(CO_3_)_2_ were the primary precursors to N-A-S-H and C-A-S-H gels. However, excessive replacement of FA with BM results in excessive amounts of Na(AlSi_3_O_8_) and CaMg(CO_3_)_2_ that cannot be fully reacted, along with insufficient active SiO_2_ and Al_2_O_3_, which inhibits the consumption of Ca(OH)_2_.

To investigate the influence mechanism of curing time on phase evolution, samples from the typical mix ratio Group R-C2 were selected for XRD testing. The curing ages were set at 7 d, 14 d, and 28 d, respectively, with results presented in Figure 12b. As curing time increased from 7 d to 28 d, the diffraction peak intensity of quartz, calcite, and other minerals decreased markedly, while the diffraction peak intensity of the hydration product AFt increased. This indicates that cement reacted with the silico-aluminum active minerals in FA and BM, and the specimens gradually developed over time, generating more hydration products. Meanwhile, the diffraction peak intensity of Na(AlSi_3_O_8_) and CaMg(CO_3_)_2_ gradually decreased, which suggests that RAP minerals may be involved in the formation of N-A-S-H and C-A-S-H gels, as inferred from the depletion of their crystalline precursors [89].

#### 3.3.3. SEM Characteristics of RFCPB

**Figure 13:**
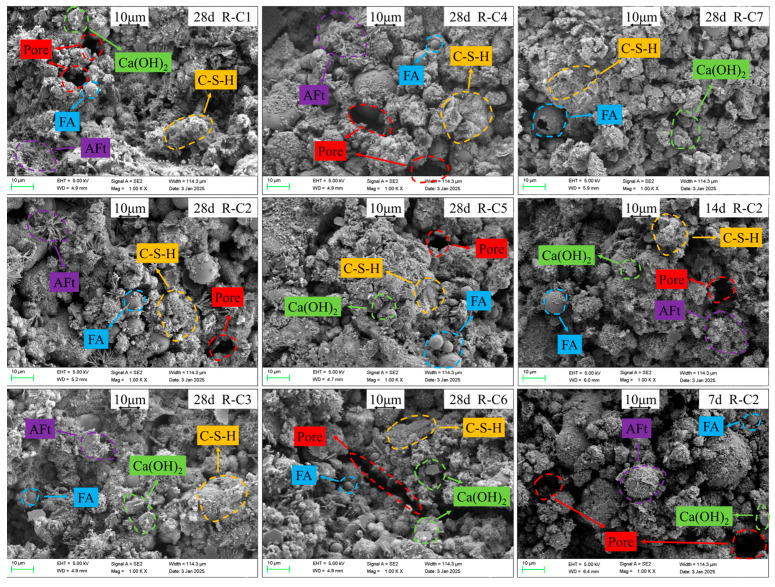
SEM results of RFCPB.

Figure 13 presents the SEM images of Groups R-C1 to R-C7 cured for 28 d and those of Group R-C2 cured for 7 d, 14 d, and 28 d.

For Group R-C2 at different curing ages, at the initial 7 d of curing, cement dominates the hydration reaction to form fine needle-like ettringite (AFt) and flake-like Ca(OH)_2_, partially encapsulating BM particles [90]. FA exists as inert spherical particles due to incomplete reaction, resulting in high matrix porosity and poor continuity of cementation. At this time, AFt exhibits a fine needle-like morphology and is more densely distributed [83]. After 28 days of curing for Group R-C2, the active glassy phase was fully activated to promote the development of C-S-H gel into a dense network structure that completely covers the mineral surface. AFt coarsened into rod-like structures and encapsulated by C-S-H, and the content of Ca(OH)_2_ decreased markedly. Hydration products effectively fill the pores, thereby achieving densification of the microstructure [91].

For Groups R-C1 to R-C7 with a curing age of 28 days, the changes in the number of pores, C-S-H, AFt, and Ca(OH)_2_ exhibit a certain trend. As the F/B ratio decreases, the coverage of C-S-H and AFt first increases and then decreases, while the number of pores and the content of Ca(OH)_2_ first decrease and then increase. This is because FA mainly contains active SiO_2_ and Al_2_O_3_, which react with Ca(OH)_2_ produced by cement hydration to form C-S-H and C-A-H gels, and partial replacement of FA with BM can further promote the hydration reaction [92].

#### 3.3.4. Pore Structure Characteristics of RFCPB

**Figure 14:**
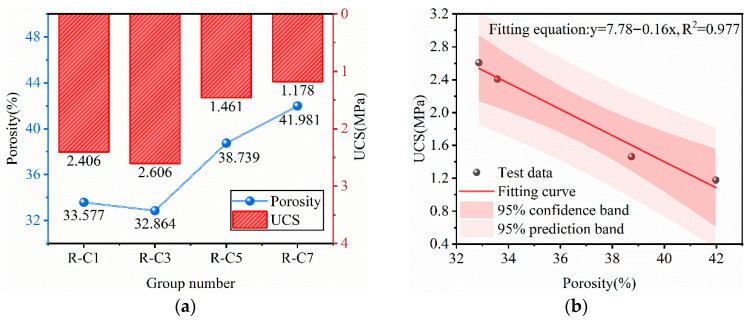
The porosity and UCS of RFCPB: (**a**) The relationship between the porosity and UCS; (**b**) The fitting relationship under different ratios.

Figure 14 reveals the quantitative correlation between the pore characteristics of RFCPBs and the UCS at 28 days of curing. Analysis indicates that excessively high porosity results in a significant decrease in UCS values [93]. Figure 14a indicates that the variations in porosity and UCS can be divided into two stages. The first stage is the slow porosity reduction zone (Groups R-C1 to R-C3): porosity decreases from 33.6% to 32.9%, representing a decrease of 2.1%, while UCS increases from 2.406 MPa to 2.606 MPa, representing an increase of 9.9%. At this stage, the micro-aggregate effect of FA dominates pore refinement, and BM synergistically optimizes particle size distribution and enhances compactness [94]. The second stage is the rapid porosity growth zone (Groups R-C3 to R-C7): porosity increases sharply to 42.0%, and UCS decreases to 1.178 MPa. Excessive BM leads to the accumulation of coarse particles to form an interlocking skeleton with voids, and the micropore filling rate decreases by 27.7% [95].

Figure 14b presents the fitted relationship between porosity and UCS for different mix ratios of RFCPBs after 28 days of curing. Analysis indicates that pore structure exerts a significant influence on the UCS of RFCPB. With increasing porosity, UCS values decrease, exhibiting an inverse proportional relationship. The following formula was derived by fitting the uniaxial compressive strength and porosity data of the backfills. The correlation coefficient of the fit is 0.977, indicating a high degree of fitting accuracy.

(5) UCS=7.78−0.16·Porosity

In the formula, *UCS* [MPa]; *Porosity* [%].

**Figure 15:**
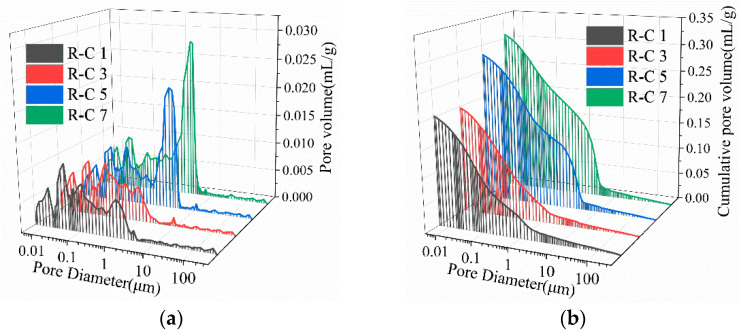
The porosity of the RFCPB: (**a**) pore size distribution; (**b**) cumulative porosity.

Figure 15 presents the pore size distribution of RFCPBs for different mix ratios after 28 days of curing. The pore size corresponding to the peak on the curve in Figure 15a is the most probable pore size, i.e., the pore size with the highest probability of occurrence [96]. Analysis reveals that the maximum pore size of RFCPBs exhibits a gradual increase. Figure 15a shows that the most probable pore size of Groups R-C1 and R-C3 at 28 d is 0.04 μm, while that of Group R-C5 is 2.06 μm and that of Group R-C7 is 2.49 μm. Figure 15b illustrates that the variation trends of Groups R-C1 and R-C4 were essentially identical, but the total pore volume of Groups R-C5 and R-C7 increased by 39.6% and 54.2%, respectively, compared with these two groups. This indicates that excessive BM content leads to pore expansion and aggregate skeleton loosening in RFCPBs, resulting in a decrease in strength. Imtiaz et al. [97] also noted that excessive addition of RAP significantly increased the number of pores in the mixture, which eventually resulted in a decrease in UCS values.

### 3.4. Leaching Toxicity Results of RFCPB

For RFCPB as a backfill material, it is essential to investigate the impact of heavy metal ion leaching from RFCPB on groundwater. The acetic acid buffer solution leaching method was used to evaluate the environmental hazard level of RFCPBs on the environment. Table 5 presents the leaching concentrations of target elements in the leachate of 28-day cured RFCPBs. Results indicate that the leaching concentrations of all seven groups meet the Grade III groundwater quality standard [98,99]. As the F/B mass ratio increases, the specific surface area of FA increases, and the leaching concentrations of heavy metal ions generally decrease. This is because the surface of C-S-H gel is capable of chemically adsorbing positively charged heavy metal ions, thus facilitating their solidification [100]. Therefore, when the F/B ratio decreases, a decrease in FA content leads to weakening of the pozzolanic reaction, reduced C-S-H production, and a reduction in specific surface area weakens surface adsorption capacity. These two factors result in a reduction in solidification efficiency [101].

## 4. Conclusions

This study systematically investigated the rheological and mechanical properties of RAP-FA cementitious paste backfill (RFCPB) under different F/B mass ratios and curing ages, and revealed the performance variation mechanisms using XRD, SEM, and other microscopic tests. Based on the experimental findings, the core conclusions are as follows:

(1) Within a reasonable dosage range, partial replacement of FA with BM does not significantly impair the workability of RFCPB slurry. RFCPB slurries exhibit typical shear thickening characteristics, and their rheological properties fit well with the Herschel–Bulkley model. The F/B ratio exerts a significant influence on the rheological parameters: as the F/B ratio decreases, the yield stress and apparent viscosity of the slurries increase significantly, while the slump and slump flow decrease. All the prepared filling slurries satisfy the technical requirements for pipeline transportation. Nevertheless, the RC-6 and RC-7 groups exhibited obvious performance deterioration; thus, only the formulations of RC-1 to RC-5 are recommended for subsequent research and application.
(2) Appropriate replacement of FA with BM synergistically improves the mechanical strength and deformation capacity of RFCPB. The 28-day UCS of RFCPB first increases and then decreases with decreasing F/B ratio, with the maximum value observed in Group R-C2. The macroscopic failure mode transitions from brittle splitting tensile failure to ductile shear failure, and the compressive deformation capacity is significantly enhanced. All groups satisfy the minimum bearing capacity requirement for coal mine goaf backfills.
(3) Active components (dolomite, albite) in BM participate in hydration reactions, promoting the formation of secondary products (AFt, C-S-H, C-A-H). These products effectively fill internal pores, refine the pore structure, and reduce total porosity, providing a microstructural basis for the improvement of UCS.
(4) Heavy metal leaching concentrations of RFCPB comply with the Grade III groundwater quality standard, verifying the environmental safety of RAP as a backfill material and providing technical support for the resource utilization of RAP.

## Figures and Tables

**Table 1:** Main chemical composition of FA, Milled material, and Cement (%).

Chemical Composition	Al_2_O_3_	SiO_2_	CaO	Fe_2_O_3_	K_2_O	MgO	TiO_2_	SO_3_	Others
BM	11.29	41.80	31.42	5.74	1.32	3.64	0.57	0.98	3.24
FA	16.52	41.01	14.03	14.47	2.33	1.52	1.08	4.17	4.87
OPC	5.53	22.36	66.08	3.46	0.62	1.27	0.59	/	0.09

**Table 2:** Test Proportions (per 1000 g of backfill slurry).

Groups	FA/BM	FA + BM, g	Crushed Material, g	Cement, g	Water, g
R-C 1	7/1	292	365	73	270
R-C 2	6/2				
R-C 3	5/3				
R-C 4	4/4				
R-C 5	3/5				
R-C 6	2/6				
R-C 7	1/7				

**Table 3:** The fitting results of filling rheological parameters in each group.

Test Number	Model	Model Relationship	$\tau_{0}$	K	n	R^2^
R-C1	H-B	$\tau =\tau_{0}+K\gamma^{n}$	12.93	0.0032	2.334	0.9994
R-C2			25.57	0.0069	2.152	0.9990
R-C3			29.27	0.0966	1.572	0.9973
R-C4			26.25	0.0802	1.610	0.9916
R-C5			39.09	0.0932	1.580	0.9970
R-C6			53.18	0.0720	1.670	0.9963
R-C7			50.40	0.0688	1.652	0.9962

**Table 4:** UCS results of RFCPB.

Sample	7d					14d					28d				
	No. of Sample (N): 5					No. of Sample (N): 5					No. of Sample (N): 5				
	UCS	SD	CV, %	95% CI	*p* Value	UCS	SD	CV, %	95% CI	*p* Value	UCS	SD	CV, %	95% CI	*p* Value
R-C1	0.624	0.035	5.64	0.580 ~ 0.668	5.53 × 10^−12^	0.885	0.058	6.52	0.813 ~ 0.957	1.35 × 10^−10^	2.432	0.198	8.14	2.186 ~ 2.678	2.17 × 10^−13^
R-C2	0.678	0.035	5.20	0.634 ~ 0.722		1.143	0.071	6.21	1.055 ~ 1.231		2.723	0.117	4.29	2.578 ~ 2.868	
R-C3	0.5423	0.0388	7.15	0.494 ~ 0.590		1.0262	0.0775	7.56	0.930 ~ 1.123		2.5819	0.1121	4.34	2.443 ~ 2.721	
R-C4	0.6376	0.0453	7.1	0.581 ~ 0.694		1.0191	0.0894	8.77	0.908 ~ 1.130		2.3887	0.1922	8.04	2.150 ~ 2.627	
R-C5	0.4022	0.0421	10.48	0.350 ~ 0.455		0.7652	0.0263	3.43	0.733 ~ 0.798		1.4703	0.0783	5.33	1.373 ~ 1.568	
R-C6	0.5268	0.0349	6.63	0.483 ~ 0.570		0.7721	0.06	7.77	0.698 ~ 0.847		1.6726	0.1072	6.41	1.540 ~ 1.806	
R-C7	0.4508	0.0291	6.44	0.415 ~ 0.487		0.7753	0.0472	6.09	0.717 ~ 0.834		1.1676	0.0964	8.26	1.048 ~ 1.287	

**Table 5:** Leaching results of RFCPB.

Element	R-C1, μg/L	R-C2, μg/L	R-C3, μg/L	R-C4, μg/L	R-C5, μg/L	R-C6, μg/L	R-C7, μg/L	Limit, μg/L
Mn	1.3	1.1	0.5	1.6	2.6	3.6	8.6	100
Zn	0.8	4.5	2.7	2.4	3.6	2.6	2.9	1000
As	1.4	1.1	0.2	3.9	3.3	4.1	2.4	10
Cd	0.6	1.2	2.0	1.0	2.4	2.0	2.4	5
Hg	0.04	0.04	0.04	0.04	0.04	0.04	0.04	1
Pb	0.9	1.7	1.2	3.4	1.8	2.6	1.5	10
Cr	0.7	1.7	1.7	3.1	4.1	4.4	6.1	50
Cu	0.3	2.5	2.1	4.1	2.1	4.3	3.4	1000
Ba	0.9	0.8	1.2	1.3	1.5	1.7	2.6	700
Ni	1.0	2.0	0.4	1.2	2.8	0.9	6.2	20
Ag	1.3	0.2	1.1	1.3	0.6	1.7	7.0	50
Se	1.2	2.0	1.6	2.4	2.7	5.3	5.9	10
Mo	0.8	1.3	1.0	0.8	4.1	2.2	11.3	70
Sb	1.6	1.7	2.7	0.6	1.2	1.2	1.2	5
Co	1.9	1.0	1.1	2.2	2.8	2.2	2.8	50

## Data Availability

The original contributions presented in this study are included in the article. Further inquiries can be directed to the corresponding author.

## Abbreviations

The following abbreviations are used in this manuscript:

RAP	Recycled asphalt pavement
CM	Crushed materials
BM	Ball-milled materials
FA	Fly ash
RFCPB	RAP-fly ash cement paste backfill
F/B	FA/BM mass ratio
H-B	Herschel–Bulkley
UCS	Unconfined compressive strength
CBC	Cement-based composite backfill
CTB	Cement tailings backfill
WTSF	Waste tire steel fiber
OPC	Cement
PSD	Particle size distribution
C_2_S	Dicalcium silicate
C_3_S	Tricalcium silicate
C_4_AF	Tetracalcium aluminoferrite
C_3_A	Tricalcium aluminate
C-S-H	Calcium silicate hydrate
N-A-S-H	Sodium-Aluminum-Silicon-Hydrate
C-A-S-H	Calcium-Aluminum-Silicon-Hydrate
CI	Confidence interval
XRF	X-ray Fluorescence
XRD	X-ray Diffraction
SEM	Scanning electron microscope
TG	Thermogravimetry
DTG	Derivative thermogravimetry
SD	Standard deviation
CV	Coefficient of variation
AFt	Ettringite
